# Differences between cotranscriptional and free riboswitch folding

**DOI:** 10.1093/nar/gkt1213

**Published:** 2013-11-25

**Authors:** Benjamin Lutz, Michael Faber, Abhinav Verma, Stefan Klumpp, Alexander Schug

**Affiliations:** ^1^Steinbuch Centre for Computing, Karlsruhe Institute of Technology, 76344 Karlsruhe, Germany ^2^Department of Physics, Karlsruhe Institute of Technology, 76149 Karlsruhe, Germany and ^3^Max Planck Institute of Colloids and Interfaces, 14424 Potsdam, Germany

## Abstract

Riboswitches are part of noncoding regions of messenger RNA (mRNA) that act as RNA sensors regulating gene expression of the downstream gene. Typically, one out of two distinct conformations is formed depending on ligand binding when the transcript leaves RNA polymerase (RNAP). Elongation of the RNA chain by RNAP, folding and binding all occurs simultaneously and interdependently on the seconds’ timescale. To investigate the effect of transcript elongation velocity on folding for the S-adenosylmethionine (SAM)-I and adenine riboswitches we employ two complementary coarse-grained *in silico* techniques. Native structure-based molecular dynamics simulations provide a 3D, atomically resolved model of folding with homogenous energetics. Energetically more detailed kinetic Monte Carlo simulations give access to longer timescale by describing folding on the secondary structure level and feature the incorporation of competing aptamer conformations and a ligand-binding model. Depending on the extrusion scenarios, we observe and quantify different pathways in structure formation with robust agreements between the two techniques. In these scenarios, free-folding riboswitches exhibit different folding characteristics compared with transcription-rate limited folding. The critical transcription rate distinguishing these cases is higher than physiologically relevant rates. This result suggests that *in vivo* folding of the analyzed SAM-I and adenine riboswitches is transcription-rate limited.

## INTRODUCTION

Riboswitches are part of the untranslated region of messenger RNA (mRNA) that reacts to specific small metabolites by conformational changes. These conformational changes modulate gene expression by terminating transcription or inhibiting translation initiation of the downstream gene (1–5). To this end, a riboswitch consists of two structural components: aptamer region and expression platform. The aptamer region is responsible for detecting and binding the ligand. The expression platform performs the desired structural reaction to the respective folded aptamer that can attenuate transcription or translation. For some riboswitches, surprisingly large ligand concentrations were found to be required for binding (6). This finding suggests that ligand binding may effectively be a slow process because conformational changes in the aptamer prohibit binding, and that there may not be enough time for metabolite binding to reach thermodynamic equilibrium. Therefore, one goal of riboswitch investigation is to determine whether a riboswitch is thermodynamically or kinetically controlled (7–9). If the control is kinetic, a crucial aspect of the structural dynamics is that folding may occur cotranscriptionally, i.e. structure formation starts while the RNA chain is elongated by an RNA polymerase (RNAP). Cotranscriptional folding can be expected to be of particular importance for riboswitches that act by terminating transcription, as in this case the regulatory decision has to be made during a short time window while the RNA chain is growing. Indeed, in a recent single-molecular study, cotranscriptional riboswitch folding and ligand binding of the *pbuE* adenine riboswitch has been directly observed *in vitro* (10) and was found to be kinetically controlled.

Cotranscriptional folding of RNA differs from free folding in two aspects: (i) the chain grows with time and thus the set of potential interaction partners for any atom in the chain evolves with time. As a consequence, the range of possible structures or folds is also time-dependent and different substructures may have different time windows for folding. (ii) Spatial restriction arises from the interaction with the RNAP. The RNAP is the machinery that reads out genetic information from DNA and synthesizes a complementary RNA strand (11–13). The nascent RNA leaves the RNAP through the exit channel which imposes spatial constraints for the emerging RNA allowing each nucleic acid one by one to leave the RNAP. Only outside the RNAP secondary structural elements can be formed. Thus, the nascent RNA strand experiences drastic spatial restrictions during transcription. This suggests that the spatial constraints introduced by the RNAP may have an influence on the folding characteristics of the riboswitch and, eventually, ligand binding.

**Figure 1.**
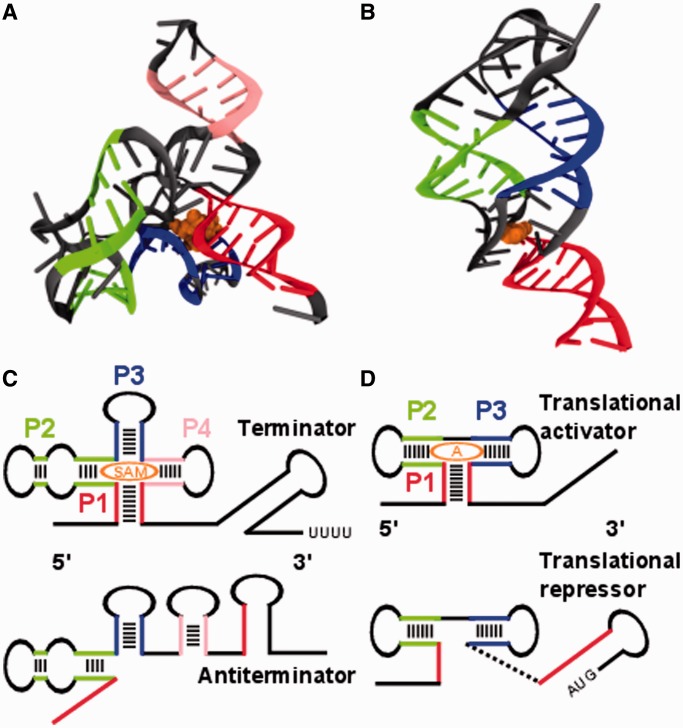
Tertiary and secondary structures of the SAM-I and adenine riboswitches in ligand bound state. (**A**) Aptamer region of the SAM-I riboswitch (PDB ID 2GIS): the colored strands indicate elements of secondary structure, helix P1 in red, P2 in green, P3 in blue and P4 in pink. The ligand is shown in orange. (**B**) Aptamer region of the *add* adenine riboswitch (PDB ID 1Y26). The same colors are used as in (A) for helices P1 to P3 and ligand. (**C**) The SAM-I riboswitch consists of two pairs of coaxially stacked helices P1 to P4 connected by a four way helical junction in its ligand bound state. Helix P1 forms in the presence of the ligand and acts as an antiantiterminator allowing the terminator (long-stem loop with downstream sequence of uridines) to fold. In this case, transcription is terminated. (**D**) The *add* adenine riboswitch exhibits three helices P1 to P3 in its ligand bound state two of which are coaxially stacked. Helix P1 forms in the presence of the ligand and prevents a translational repressor (initiation codon paired in long-stem loop) from forming.

Computational and analytical methods complement experimental studies to systematically refine our understanding of RNA folding (14–18). Atomically resolved simulations provide microscopic insight into the folding behavior of riboswitches. Straightforward molecular dynamics (MD) simulations with explicit solvent are computationally highly demanding, thus they are limited to short simulation times on the order of hundreds of nanoseconds (19). As riboswitch folding occurs on the order of seconds, such simulations would exceed present day computational resources by several orders of magnitude. Moving beyond these limitations, this study focuses on two structure-based computational methods to investigate the cotranslational folding of two exemplary riboswitches (Figure 1).

As a first approach, we propose a simulation protocol based on structure-based models (SBMs), similar to an earlier study of free riboswitch folding (20). SBMs employ a potential that is based on the native fold of a biomolecule. Motivated by energy-landscape theory (21,22), this model exhibits a smoothly funneled energy landscape dominated by native interactions. The coarse-graining of this model allows us to reach the biologically relevant time scales of RNA folding with comparably moderate computational effort (23,24) while still being atomically resolved and dynamic.

**Figure 2.**
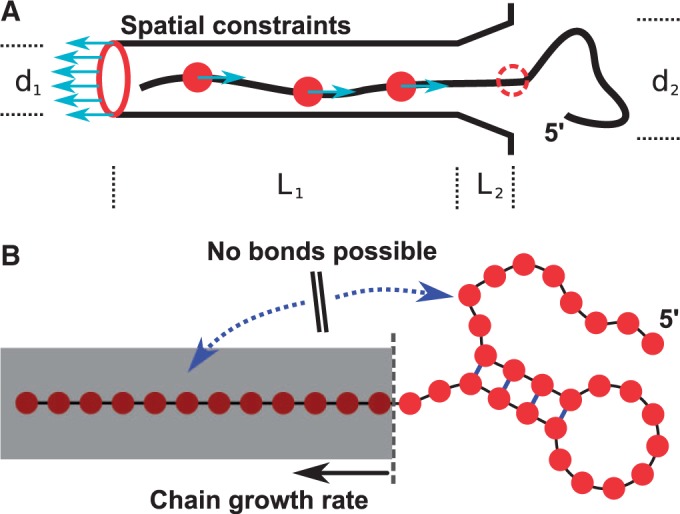
Schematics of the setups for SBM and kinetic MC simulations. (**A**) Schematics of the setup for an SBM simulation. The tube with a funnel-like exit region composed of a helix of SBM atoms surrounds the stretched RNA. The tube/atoms are positioned on a helix with a diameter *d_1_* of 20 Å and a length *L_1_* of 1100 Å to contain the whole stretched RNA strand. The exit funnel has a length *L_2_* of 40 Å and an outer diameter *d_2_* of 30 Å. These are the spatial constraints that prevent folding before the riboswitch has left the RNAP. Forces acting between the rear end of the tube (red ring) and every tenth nucleotide (red circles) extrude the RNA strand out of the tube with a constant rate. Whenever a nucleotide leaves the tube, it is released of its acting force and therefore free to fold mimicking the natural sequential transcription process. (**B**) Schematics of the kinetic MC method. The kinetic MC method grows the RNA chain with a constant rate allowing more and more base pairs to form or open in the available sequence.

In the present model, the stretched RNA strand is forced out of a flexible tube with a funnel-like exit region emulating the extrusion of nascent RNA out of the RNAP (Figure 2A). Thereby, the force is distributed over a number of residues while they are inside the tube. Every segment of the strand that leaves the tube is released of the force and can fold freely. In addition, we study the cotranscriptional folding of secondary structure using kinetic Monte Carlo (MC) simulations. Unlike earlier MC studies (25,26), but similar to the SBM, we employ a recently proposed approach that incorporates native secondary structural information into our MC simulations (27). For the present study, we modify this approach to include RNA growth, which is mimicked by sequentially enlarging the subset of contacts that can be formed during the simulation. Only contacts between pairs of nucleic acids that have both been already transcribed can be formed (Figure 2B). The RNAP’s transcript elongation rate is known to be variable over the range of about one order of magnitude (∼15 to 80 nucleotides per second, nt/s) (11). The different elongation rates are accomplished by pausing of transcription which is regulated by a variety of mechanisms (28–30). The kinetic MC approach also allows us to simulate the competition in folding of the alternative structures by including two sets of ‘native’ contacts. We use this approach to compare free and cotranscriptional folding of the competing structures and to estimate the stabilizing effect of the ligand on the riboswitch aptamer structure.

## MATERIALS AND METHODS

### System of interest

We investigate the aptamer regions of two riboswitches with different switching behavior and regulatory functions. The S-adenosylmethionine (SAM)-I riboswitch from *Thermoanaerobacter tengcongensis* is an ‘off’ switch that regulates transcription. The sequence we investigate consists of 94 nt (PDB: 2GIS) (31) (shown in Figure 1A, detailed base pairing in Supplementary Figure SI-1). The 94 nt are arranged in a four-way helical junction with two pairs of coaxially stacked helices termed P1–P4. Their stems consist of 8, 7, 6 and 5 bp, respectively. The metabolite SAM binds to a binding pocket between P1 and P3. In addition, we discuss the adenine riboswitch from *Vibrio vulnificus*. This riboswitch is an ‘on’ switch that modulates translation initiation and consists of 71 nt arranged in a three-way helical junction PDB ID 1Y26 (32) (shown in Figure 1B, detailed base pairing in Supplementary Figure S1). Three helices P1 to P3 exhibit stems consisting of 9, 6 and 6 bp, respectively. The ligand binds between the two coaxially stacked helices P1 and P2.

To investigate how folding is affected by transcription we focus on understanding how the folding order of the substructural elements depends on the transcription rate. We study two folding scenarios, the free folding of the whole structure and the cotranscriptional folding of the RNA. We choose the number of formed base pairs as the reaction coordinate for our analyses. This choice allows us to sample over stochastically generated trajectories and projects the folding progress to a globally comparable variable. The observables we are interested in are the numbers of formed base pairs within substructural elements. Specifically we look at the stems of local and nonlocal helical loops defined by the given secondary structure of the riboswitch. Formation of a base pair in the SBM is considered as being achieved whenever >50% of the native interatomic contacts between two bases are formed. Further investigations show that the folding characteristics are stable with regard to the actual choice of this threshold (Supplementary Figure S2). The choice of reaction coordinate also facilitates the comparability with the dynamic MC method, where the number of base pairs is the natural reaction coordinate. Having determined reaction coordinate and observable for our simulations we require suitable methods to model transcription as a biomolecular process. The two different employed simulation methods require different setups to model the systems of interest.

### Native structure-based MD

MD simulation technique solves Newtonian equations of motion for a system of interest by numerically integrating the equations over time. The system of interest is introduced by a characteristic potential from which the forces in the equations of motion are derived. According to the theoretical framework of energy landscape theory and the principle of minimal frustration, evolutionary pressure selects a protein’s energy landscape to be smooth (i.e. expressing only minimal local roughness) with a funnel-like shape biased towards the native state (21,22). This results in efficient folding ruled by cooperative (native) interactions, which has also been shown for noncoding RNA (33). Native SBMs employ the ideal case of a perfectly smooth, funneled energy landscape where only interactions of the native conformation are taken into account. An all-atom formulation of the structure-based potential (20,34) reads as
(1)
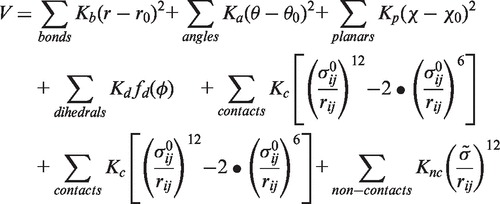

where the dihedral or torsional angle potential is given by
(2)
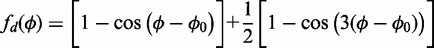

And *K_b_*, *K_a_*, *K_p_*, *K_d_*, *K_c_* and *K_nc_* are the corresponding force constants. The parameters *r*_0_, *θ*_0_, *χ*_0_, *ϕ*_0_ and 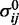 are taken from the native structure and 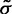 is a global exclusion radius. Accordingly, the potential has its minimum at the native conformation. The information of bonded interactions (bonds, angles, planar dihedral and proper dihedral angles) is complemented by contact information that is introduced via Lennard–Jones terms in 1. This information is aggregated in the contact map of a biomolecular structure. We use a shadow map as contact map (35) that regards atoms in contact within 6 Å radius as long as they are not shadowed by atoms within the connecting line. Nucleic acids are allowed to form contacts between neighboring residues in order to be able to model stacking interactions. All other possible pairings of atoms are assigned to repulsive Lennard–Jones terms that are characterized by the exclusion radius 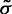. The SBMs for our simulations are generated by the SMOG webserver (36).

The introduced force constants are homogenous and normalized with respect to the system size and number of contacts. We ran our simulations with the Gromacs software package (37) in reduced Gromacs units, which do not possess direct physical time and temperature scales (21,22,38,39). Therefore they need to be introduced by comparison of observables with experiments or empirical force–field simulations. To calibrate the relative temperature scales with empirical force-fields, we perform an all-atom MD simulation based on the AMBER99 force field with TIP3P and counter ions (40). We simulate 1 μs at a reference temperature of 300 K (26.85°C) and compare the spatial root mean-square fluctuations (RMSF) (41) of individual residues with SBM simulations at various temperatures. The best agreement is obtained for SBM simulation at a temperature of 90 (reduced Gromacs units, Supplementary Figures S10 and S11). The riboswitch starts losing structural stability in the base pairs above 90 (reduced Gromacs units) and folding pathways become less distinct. Below 90 the order of folding events does not change between 62 and 90 (reduced Gromacs units). The lower temperature, however, accelerates folding by about one order of magnitude and reduces computational effort significantly which allows enhanced sampling.

We run 180 free-folding simulations at the temperature of 62 (reduced Gromacs units) to get an estimate of the folding time that can be compared with experimental results (42). In order to gain an estimate for the simulation folding time, the root mean-square deviations (RMSD) with respect to the native fold of each simulation frame are extracted from the trajectory. As soon as an RMSD drops below a threshold of 3 Å the corresponding frame is considered as a folding event. A histogram of these 180 folding events yields a slightly asymmetric distribution whose maximum we regard as an estimate for the folding time (Supplementary Figure S3). The comparison with an experimental value for the folding time of an adenine riboswitch (42) yields 20 nt/s for our smallest extrusion rate of 0.0025. The simulations are performed at temperatures of 62 and 90 (reduced units, 90 reference temperature). A time step of 0.001 is used for the extrusion simulations and 0.002 for the free-folding simulations. The temperature is introduced and kept constant via Langevin dynamics with a coupling constant of 1. In the extrusion scenario, we apply a constant velocity pull option with different constant rates, ranging from 0.0025 to 0.1 (see also exemplary Supplementary Movie S1).

### Kinetic MC method

RNA is modeled as a linear chain of bases (*b_1_,b_2_, … ,b_N_*) where *b_i_* = A, C, G or U. A set of base pairs *(b_i_, b_j_)* defines a secondary structure of the RNA. The free energy of a given secondary structure is calculated using empirical models and parameters commonly used in RNA secondary structure prediction (43). This parameterization comprises sequence dependent energy values for stacks of two subsequent base pairs, mismatches, short loops and bulges. Larger loops and loops connecting multiple helices are parameterized depending on their length, symmetry and number of outgoing helices. A total free energy is then approximated by the sum of all structural motifs 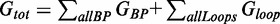. Similar to the structure based approach used above structural information is incorporated into the RNA model. A list of contacts that are closed in the native structure is provided and the base pairing interactions are restricted to those listed. In the simulations with two competing structures, corresponding to two free energy minima, the list includes native contacts of both structures. Therefore some contacts in the list are mutually exclusive. We utilize a MC simulation scheme with Metropolis rates where basic moves are the closing and opening of single native base pairs. A move is accepted with a probability 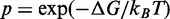 where 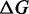 is the free energy difference of the secondary structures before and after the move. Moves that lead to 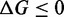 are always accepted. To fix the simulation timescale we calculate the average transition time from the unfolded to the folded state of the adenine riboswitch aptamer region (Supplementary Figure S4). By comparison with experimental results (42) we obtain the relationship of 1 s corresponding to (1.2 × 10^5^) MC steps.

## RESULTS

### Folding of riboswitch aptamers in SBM simulations

The nascent RNA strand is generated by pulling apart both ends of the native structure to a linear chain of maximal length in a SBM simulation. The RNAP is modeled by bonded beads positioned at helically parameterized coordinates of a hollow cylinder that is long enough to contain the respective stretched RNA strand. The exit region is modeled at the end of the hollow cylinder by a continuation of bonded beads at positions of a funnel-like structure with a rim. The beads connected via bonds have the uniform exclusion radius of a SBM atom (see ‘Materials and Methods’ section), which creates spatial constraints of hard spheres in the form of a tube (Figure 2A). The stretched RNA strand is then placed inside the tube and a SBM is generated for the combined structure. The SBM contains the geometry of both individual structures without topological interactions. The extrusion process is modeled by a force that acts between the rear end of the tube and every tenth nucleic acid of the nascent strand, which is thereby driven out of the tube. As soon as a residue leaves the tube it is released from its acting force and therefore, in principle, free to form nonlocal contacts and fold.

**Figure 3.**
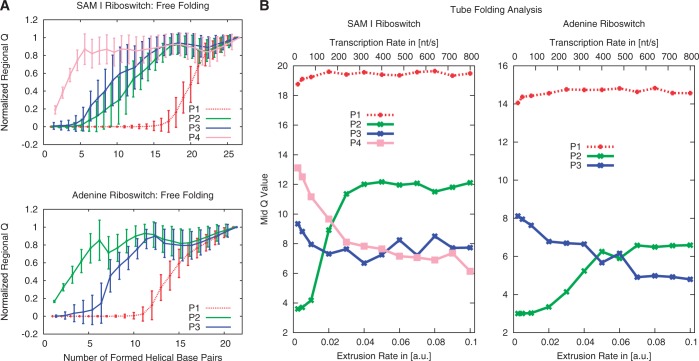
Folding analysis based on SBM simulations. (**A**) Folding pathways of the SAM-I and adenine riboswitch for free folding. The plots for each riboswitch are based on 180 folding trajectories, starting from a stretched RNA strand. The mean values and standard deviations are plotted. We can derive the folding order by condensing a substructure’s curve in a single value representing the mid *Q-*value. (**B**) Folding events of substructural elements over the extrusion rate. A folding event is characterized by the number of formed helical base pairs at a normalized regional *Q*-value of 0.5, the mid *Q*-value. The regional *Q*-value data are gathered from 80 trajectories for each extrusion rate. The nonlocal helix P1 ties up both ends of the sequence and, therefore, folds last in both riboswitches. Both riboswitches fold in order of appearance in the limit of slow extrusion rates. We investigate a wide range of extrusion rates to cover the natural range of transcription rates. An extrusion rate of 0.0025 corresponds to an estimated transcription rate of ≈ 20 nt/s.

For both riboswitches, 180 free-folding simulations and 80 simulations at each of 12 different extrusion rates are performed in this study (see ‘Materials and Methods’ sections). Figure 3A shows the mean values and standard deviations of the normalized regional *Q*-values for each helical stem over the total number of formed contacts during free folding. The regional *Q*-value represents the number of formed base pairs within the respective helical substructure. Mean value and standard deviation illustrate the approximate Gaussian distribution of *Q*-values within a contact bin. The SAM riboswitch exhibits immediate folding of helix P4, followed by P3 and P2 with relatively small difference. The nonlocal helix P1 constitutes the distinct end of the folding process by tying up both ends of the RNA strand, in agreement with earlier simulations (20). The adenine riboswitch starts folding with helix P2, followed by P3 and concluded by the nonlocal helix P1. The transitions can be characterized by the number of formed helical base pairs at which the normalized regional *Q*-value = 0.5. This procedure yields mid points of the regional folding characteristics, respectively, condensing each curve in Figure 3A into a single value—the mid *Q*-value. Mid *Q*-values for all 12 extrusion rates are shown in Figure 3B summarizing our investigations of cotranscriptional RNA folding by means of the SBM. We see an influence of the extrusion rate on certain sub-structural elements: In the SAM riboswitch, the folding order of P2 and P4 is reversed at a certain critical rate, whereas, in the adenine riboswitch both P2 and P3 do not depend that strongly on the rate. The formations of P2 and P3 in the SAM riboswitch and in the adenine riboswitch are simultaneous for a wide range of rates within the scope of the *Q*-values uncertainties. The nonlocal helix P1 is relatively independent of the extrusion rate in both riboswitches. In both cases, transitions in the folding order occur at extrusion rates (>100 nt/s) that are beyond the range of physiologically relevant transcription rates. The time scale of folding was set by comparison of the free-folding time of the adenine riboswitch with the experimental value (Supplementary Information). Based on that observation, the free-folding case can be distinguished from the transcription-rate limited case.

### Folding of the aptamer region with the MC method

In addition to the SBM simulations, we employ a kinetic MC simulation scheme where the elementary steps are the opening and closing of individual base pairs. Similar to the SBM *ansatz*, structural information is incorporated into the model and simulations concentrate on the native secondary contacts. Here we utilize two different simulation setups. On one hand, we simulate the dynamics of free folding of the full-length chain without further constraints. On the other hand, to mimic cotranscriptional folding the RNA is divided into a free (already transcribed) and a confined (not yet transcribed) part. Formation of a base pair is only possible if both participants belong to the free part, while those that are confined cannot form contacts. RNA chain growth corresponds to a shift of the boundary between these regions.

**Figure 4.**
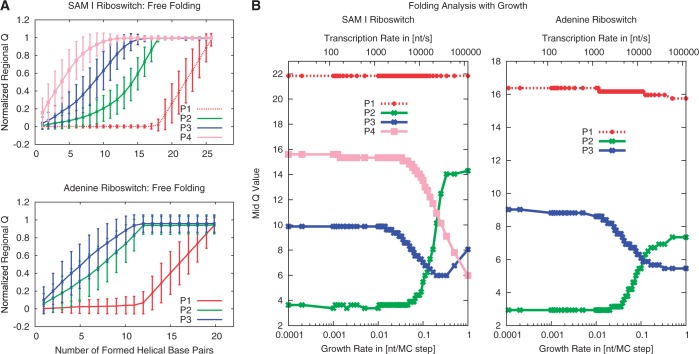
Folding analysis based on Kinetic MC simulations. (**A**) Fraction of base pairs formed in each helix (normalized regional *Q*-value) as a function of the total number of formed base pairs for free folding of the adenine and SAM-I riboswitch secondary structures. (**B**) Folding of each helix over the chain growth rate for the SAM-I and adenine riboswitches. Folding events are characterized by the number of base pairs formed in the whole structure when a normalized regional *Q*-value of 0.5 is reached (mid *Q-*value, Figure 3B).

In the free-folding case we start with a conformation where no base pairing contacts are present and observe a rapid formation of secondary structure over the course of our simulations. For both secondary structures, folding has a distinct order. Figure 4A shows the mean *Q* for each of the stems as a function of totally formed base pairs. The folding order in the SAM riboswitch is the same as above—P4 folds first, followed by P3, P2 and P1, where the latter starts folding only after the first three have folded. Similarly the adenine riboswitch folds the helices P2 and P3 first, followed by the helix P1, which brings the two ends of the RNA together. While in the MC simulations the two stems P2 and P3 fold simultaneously the SBM simulations show P2 to fold first. This suggests that tertiary interactions, which are not included in the MC approach, play a role in determining the folding order in this case.

Next, we perform the simulations with RNA growth, where we start with one free base and stochastically move the boundary with a rate corresponding to the chain growth rate. Because the MC simulations are computationally much less demanding than the SBM simulations, we can vary the rates over a wider range. Figure 4B shows the dependence of the folding events on the chain growth rate (as above characterized by the mid *Q*-value). In the slowly growing regime the secondary structure elements form in the order of their appearance (P2, P3, P1 for the adenine riboswitch and P2, P3, P4, P1 for the SAM riboswitch). This order is different from the one observed in the free-folding case above, which is recovered in the fast growing regime. The folding order depends on the chain growth rate in qualitatively the same way in both SBM and MC simulations. However, the transition to free folding occurs for larger transcription velocities in MC than in SBM (≈0.1 nt/MC-step or 10^4 ^nt/s, with the conversion of MC steps to seconds again by comparison with the experimental free-folding time, see also Supplementary Figures S8 and S9). In both cases, substructures fold in the order of appearance throughout the physiological range of elongation velocities.

### Folding of the complete riboswitch with the MC method including competing minima for the aptamer region and the expression platform

The simulations so far have described only one structure of the riboswitch, corresponding to a single minimum in the energy landscape, namely the aptamer region, which is formed in the presence of high ligand concentrations. We now investigate how the inclusion of the competing expression platform influences the folding behavior of the aptamer by extending the sequences of both riboswitches to include the sequence parts that build the competing alternative structure (see sequences and base pairing in Supplementary Figure S5). For the SAM-I riboswitch we include the antiterminator (AT) hairpin which consists of 12 bp (31) and for the adenine riboswitch we add the 18-bp hairpin that blocks the Shine–Dalgarno (SD) sequence for translational repression (TR) (32). The list of ‘native' base pairs now includes all base pairs formed in the competing structures. For both riboswitches, not all ‘native’ base pairs can form simultaneously, as the newly added alternative 3′ structure competes with the nonlocal helix P1 which would close the multiloop structure of the aptamer. Empirically, low metabolite concentrations lead to formation of AT/TR, while high concentrations stabilize the multilloop region including P1.

**Figure 5.**
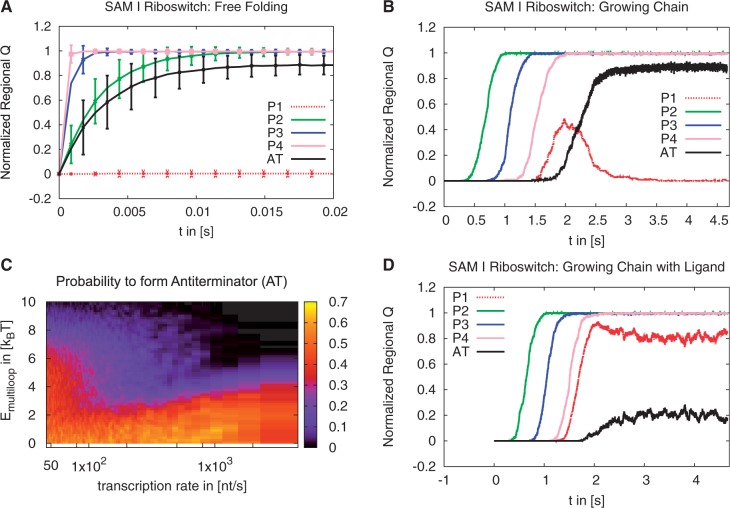
Folding analysis of the extended sequence of the SAM-I riboswitch using Kinetic MC simulations. (**A**) Normalized regional *Q*-value as a function of time for free folding. (**B**) Normalized regional *Q*-value as a function of time for the chain growing at a transcription rate of ≈ 50 nt/s. (**C**) Probability (color coded) to find AT formed at the time when the complete chain is fully grown as a function of the transcription rate and 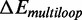. (**D**) Normalized regional *Q*-value as a function of time for the chain growing at a transcription rate of ≈ 50 nt/s. Here the multiloop is stabilized by 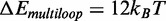 to mimic the effect of the ligand.

We follow our kinetic MC simulation protocols and first simulate free folding of the complete sequences without adding terms for the ligand (Figure 5A for the SAM-I riboswitch and Supplementary Figure S6A for the adenine riboswitch). In the SAM-I riboswitch the helices P2, P3, P4 and the AT fold very rapidly on a timescale of milliseconds. The nonlocal helix P1 does not form. Instead the AT is formed. Similarly in the adenine riboswitch the helices P2, P3 and the TR form simultaneously while the nonlocal helix P1 remains unformed. Here the structure that sequesters the SD sequence is formed. Thus, in the free-folding setup we see that for both riboswitches the newly added hairpin out-competes the multiloop structure of the aptamer. This is the expected behavior for low ligand concentration, which empirically favors formation of AT/ TR. This can be rationalized energetically as the formation of AT/ TR or P1 is both stabilized by considerable base pairing. In contrast, formation of the nonlocal helix P1 has to overcome the additional entropic penalty of forming the multiloop structure.

Next, we simulate the folding of growing chains with varying chain growth rates. In agreement with our results for the aptamer-only folding, we observe formation of substructures in the order in which they are grown. This order differs from the free-folding scenario. After equilibration the final structure, however, is the same as in the free-folding case: a structure that includes the AT/ TR and all local helices of the aptamer, but not the multibranch loop held together by the nonlocal helix P1. We find two main pathways, dependent on the chain growth rate. For fast growth (transcription rates >200 nt/s), which exceeds the physiologically possible rates, P1 does not form before the competing structure is grown and the competing structure folds rapidly (Supplementary Figure S7). At rates closer to the physiologically relevant range (transcription rates < 100 nt/s) P1 forms before the competing structure is grown and then ‘backtracks’ (44), i.e. helix P1 opens to allow formation of the more stable alternative conformation (Figure 5B), which is the desired physiological behavior in the absence of ligands. We also find backtracking in the adenine riboswitch, but with slower dynamics (≈20 s compared with 1 s for the SAM-I riboswitch). Rapid backtracking is essential for the SAM-I riboswitch’s function as a regulator of transcription termination, but may not be required for translational regulation by the adenine riboswitch (discussed in the Supplementary Data, in particular Supplementary Figure S6C).

So far, our simulations of competing conformations without ligands suggest that for the aptamer to fold and remain closed in the presence of the ligand, the multiloop structure needs to be stabilized by the ligand. The crystal structures of the aptamers in the presence of the ligand (31,32) show that, in both cases, the ligand connects the nonlocal helix P1 to other helices within the aptamer and induces tertiary contact formation. These observations suggest that the ligand indeed stabilizes the multiloop structure. At high ligand concentrations P1 formation must out-compete the alternative structure, which is the desired switching behavior. We therefore mimic the effect of a bound ligand by modifying the free energy *G* by an energy term 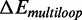 that reduces the initiation penalty of a multiloop and vary it on a scale of a few *k_B_T*. Different ligand concentrations can be simulated by varying this energy stabilizing the multiloop region, representing cooperative stabilizing energies from ligand binding. For the SAM-I riboswitch, we start with just one free nucleotide and simulate the folding of a growing chain. We calculate the probability to find the competing AT conformation at the point in time where the complete chain has grown, varying the chain growth rate and 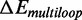. If the energy modification is small, we recover the folding behavior as in the absence of ligands. With increasing 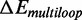 however, we find regimes where the closed aptamer conformation is stable and therefore the competing structure not formed (Figure 5C and D). Our simulations suggest that a stabilizing energy of ∼7 *k_B_T* for the SAM-I riboswitch is sufficient for robust formation of the closed aptamer at slow transcription rates (≈50 nt/s). Similarly, we see a stabilizing effect of the ligand on the adenine riboswitch’s P1 helix (discussed in the Supplementary Information).

## DISCUSSION

The folding of riboswitches is a complex biomolecular process that involves two competing (meta-) stable structures, the binding of a ligand, electrostatic effects and the growth of the RNA chain, in the case of cotranscriptional folding. The latter is physiologically crucial in particular for riboswitches that control transcription termination. Moreover, these processes take place on a relatively long time scale of the order of seconds. All these issues pose considerable computational challenges for simulations of riboswitch folding. In this study, we have combined two different approaches, SBM simulations and a kinetic MC approach, to address some of these challenges and to simulate cotranscriptional folding of two exemplary riboswitches, the adenine and SAM-I riboswitches.

Both methods allow us to simulate the dynamics of riboswitches on the second time scale, which exceeds today's computational capabilities for standard MD simulations by more than three orders of magnitude. SBM simulations offer computationally tractable implementations that yield atomically resolved dynamic trajectories of riboswitch folding over the physiological relevant time scale by coarse-graining the interactions. They are an established *ansatz* in protein folding based on energy landscape theory (21,22). For RNA the energy landscape is more rugged, but the general concept of tertiary (23,34) or secondary (27) structure-based simulations is justified by investigations of interaction networks that guide RNA folding (33) motivating a picture of an overall funneled energy landscape for structured RNA.

We have used both SBM simulations and kinetic MC to simulate free and cotranscriptional folding of ribosowitch aptamers into the configuration that, in their functional context, is attained in the presence of high ligand concentrations. The two competing conformations that represent the decision-making process have been incorporated in the kinetic MC simulations. This situation corresponds to an energy-landscape with two funnel-like minima. In addition, we proposed an implicit model for ligand binding in kinetic MC that mimics additional tertiary interaction induced by the ligand by lowering the energetic penalty of a multiloop structure. In contrast, including the second conformation in the SBM simulations is a nontrivial task (38,39,45) that remains unresolved. This task requires experimentally resolved tertiary structures of the competing conformation that are currently not available for the SAM-I or adenine riboswitches.

Both approaches we use do not treat electrostatic effects explicitly and assume sufficient concentrations of stabilizing ions (46). Explicit electrostatics will be required to study the effects of varying concentrations of specific ions such as magnesium, which we did not attempt here. Doing so will require additional computational effort. For SBM, electrostatic effects are typically subsumed in the Hamiltonian without explicit electrostatic term (20,34), but explicit SBM simulations focusing on electrostatic effects have been realized recently by the Debye–Hückel approximation (47,48). Likewise, the parametrization of kinetic MC is not *a priori* designed to feature electrostatic interactions explicitly (43).

The transcription rate directly defines a time window during which a ligand can bind to the riboswitch and influence its fold before the termination decision is made. Comparison of this time window with the time needed by the riboswitch-binding site to reach thermodynamic equilibrium with the metabolite determines whether a riboswitch is under kinetic or thermodynamic control. This is experimentally investigated by ‘globally’ measuring dissociation constants of ligand binding *in vitro* and comparing to required metabolite concentrations for 50% termination efficiency *in vivo*.

Our simulations add microscopic insight and ‘map’ riboswitch folding in two exemplary cases. Pending on transcription progress, the riboswitch offers the metabolite a ‘growing’ binding site with a time-dependent binding affinity. A binding pocket composed by aptamer parts close to the 5′-end effectively increases the time slot available for ligand binding compared to a binding pocket with significant parts close to 3′. Considering transcription times of typically seconds for both the aptamer and expression platform, this accounts to a significant difference compared to a fully-transcribed free-folding riboswitch.

### Summary

Riboswitches, which are located at the 5′-end of mRNAs, play a crucial role in gene expression by regulating the transcription or the translation of these mRNAs. A deeper understanding of possible influences on the involved mechanisms will facilitate insights in gene regulation, evolutionary processes or the RNA world hypothesis (49). The presented work focuses on the modeling of nascent riboswitch strands during transcription and the resulting folding pathways. We employ simulation protocols based on two different coarse-grained approaches that emulate spatial constraints of the RNAP and sequential release of the riboswitch aptamer region: a homogenized, minimally frustrated force field based on the systems native tertiary structure and a kinetic MC method based on transitions weighted by the free energy benefit from secondary structure formation. Our simulation results, which are consistent between the two simulation techniques, give a more detailed insight in cotranscriptional riboswitch folding. Folding of a SAM-I and an adenine riboswitch occurs in a transcription-rate limited order different from free folding with a critical transcription rate differentiating both scenarios. This rate, however, is too high to be of physiological relevance. The picture that emerges from our two exemplary cases of cotranscriptional riboswitch folding is surprisingly simple and concurs with recent experimental findings in single molecule measurements (10). Substructures fold in the order in which they are transcribed. However, once a competing structure is transcribed, it may invade the previously folded structure unless that one is stabilized by the bound ligand. The explicit inclusion of ligand binding (to enable varying ligand concentrations) and the implementation of the competing structures in the SBM simulations will be future refinements of our approach. Going beyond cotranscriptional folding of our specific riboswitches, the simulation protocols can be adapted to provide microscopic insight into the folding of other structured RNAs and, slightly modified, also allow simulating cotranslational protein folding.

## SUPPLEMENTARY DATA

Supplementary Data are available at NAR Online, including [50].

## FUNDING

The Helmholtz Association within the ‘Impuls- und Vernetzungsfond’. Cluster resources for our simulations were provided by the bwGRiD as part of the National Grid Initiative. Funding for open access charge: We acknowledge support by Deutsche Forschungsgemeinschaft and Open Access Publishing Fund of Karlsruhe Institute of Technology.

*Conflict of interest statement*. None declared.

## Supplementary Material

Supplementary Data
