# Supplementary material for: Differences between cotranscriptional and free riboswitch folding
Source: Nucleic Acids Res. 2013 Nov 25;42(4):2687–96. doi: 10.1093/nar/gkt1213 (PMC3936736; doi:10.1093/nar/gkt1213)
Supplement: Supplementary Data [file supp_42_4_2687__index.html]

Differences between cotranscriptional and free riboswitch folding — Differences between cotranscriptional and free riboswitch folding — Supplementary Data 

# Differences between cotranscriptional and free riboswitch folding

## Supplementary Data

files

**Files in this Data Supplement:**

- Supplementary Data - pdf file
- Supplementary Data - mpg file
