# Supplementary material for: Differences between cotranscriptional and free riboswitch folding
Source: Nucleic Acids Res. 2013 Nov 25;42(4):2687–96. doi: 10.1093/nar/gkt1213 (PMC3936736; doi:10.1093/nar/gkt1213)
Supplement: Supplementary Data [file supp_gkt1213_nar-01561-r-2013-File007.pdf]

Computational Analysis of Co-transcriptional Riboswitch Folding

Benjamin Lutz *[1,2], Michael Faber[3], Abhinav Verma[1], Stefan Klumpp[3] and Alexander Schug[1]

[1]*Steinbuch Centre for Computing, Karlsruhe Institute of Technology, Karlsruhe, Germany*
[2]*Department of Physics, Karlsruhe Institute of Technology, Karlsruhe, Germany*
[3]*Max Planck Institute of Colloids and Interfaces, Potsdam, Germany*

October 14, 2013

1 Riboswitches

Our study focuses on two riboswitches, the SAM-I riboswitch (PDB ID 2GIS) and the adenine *add* riboswitch (PDB ID 1Y26). The secondary structures for both the riboswitches are schematically shown in Fig. 1.

2 Influence of Base Pair Contact Ratio in SBM Simulations

In the course of SBM simulation evaluation we have investigated the influence of a chosen contact ratio threshold on the mid Q value. We observed robust positions of the substructural characteristics for a wide range (0.4 - 0.8) of thresholds, see Fig. 2.

3 Folding Times

In order to compare our simulations with the experimental data, a physical time scale estimate for simulations is required. We have introduced an estimate of the natural time line by comparison with experimental data of free folding events of the adenine riboswitch.

*Corresponding author: benjamin.lutz@kit.edu

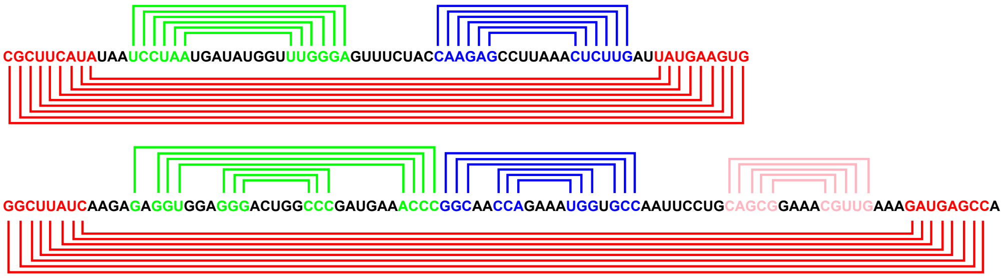

Figure 1: Secondary structure schematics of the SAM-I (bottom) and the adenine *add* (top) riboswitch in their respective ligand bound conformations.

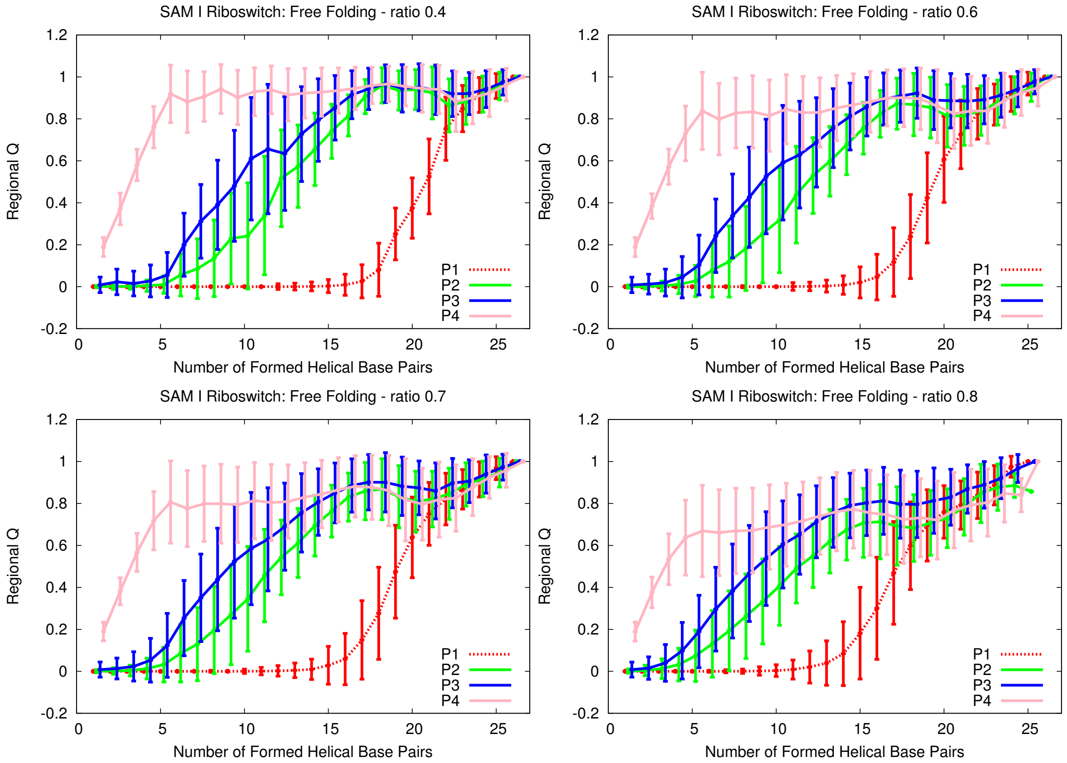

Figure 2: Study of the influence of the chosen ratio threshold on the helices' folding transitions.

3.1 SBM Simulations

We derived histograms of instances where the riboswitch is folded with a threshold RMSD (3 Å) for the first time in all 180 trajectories (see Fig. 3). The maximum of this distribution is then compared with the experimental folding times which gives us an estimate of 4000 GROMACS/SBM seconds per second in realtime .

3.2 Kinetic Monte Carlo Method

In order to estimate the time scale for Kinetic MC simulations, the mean folding time in Kinetic MC steps ($\simeq 1.5 \times 10^5$ MC Steps) is compared to experimental folding rates. This suggests that 1 sec corresponds to 1.2×10^5 MC steps. The corresponding distribution is depicted in Fig. 4.

3.2.1 Competing Structures and Ligand Model

The secondary structures of the competing conformations are shown in Fig. 5. The inclusion of competing structures and our ligand model are presented in the main text together with our results for the SAM-I riboswitch. In Fig. 6 we show the corresponding analysis of the adenine riboswitch. Free folding simulations (Fig. 6A) of the complete sequence show that the helices P2, P3 and TR are formed rapidly while the non-local helix P1 does not form. Therefore, as expected, the structure sequestering the Shine-Dalgano sequence outcompetes the aptamer at zero ligand concentration. Co-transcriptional folding, similar to the SAM riboswitch, exhibits two pathways which relax for long times in the free folded conformation. At very fast transcription (transcription rates >400 nt/s) P1 does not form at all (Fig. 6B). For physiologically relevant rates, however, P1 forms before the TR is transcribed and opens

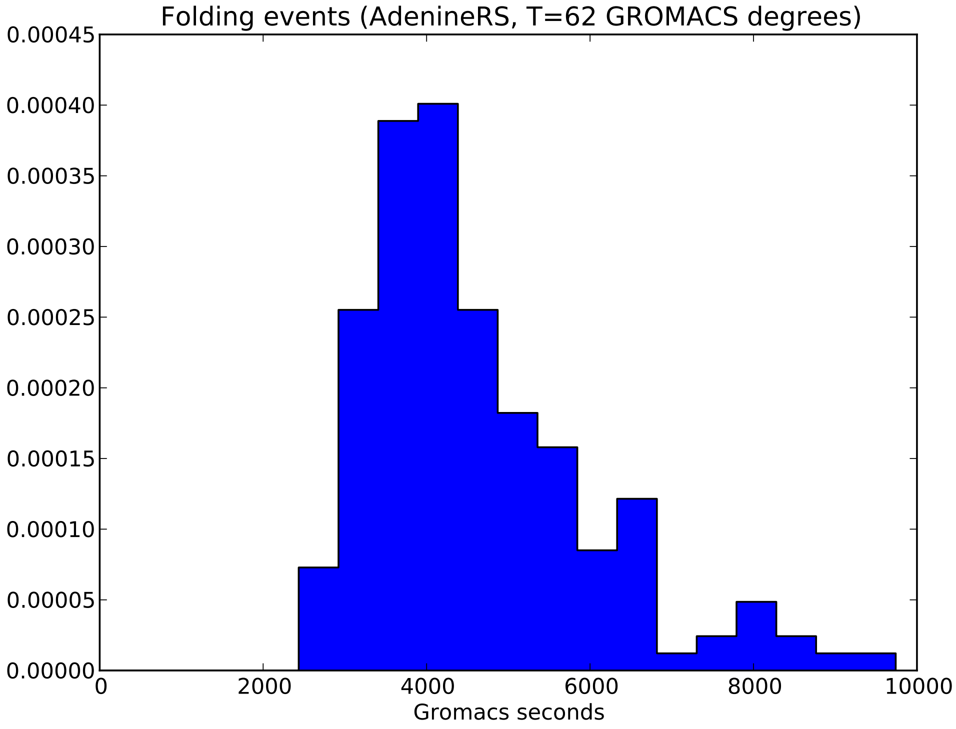

Figure 3: Normalized histogram of folding times in SBM simulations for the free folding adenine riboswitch.

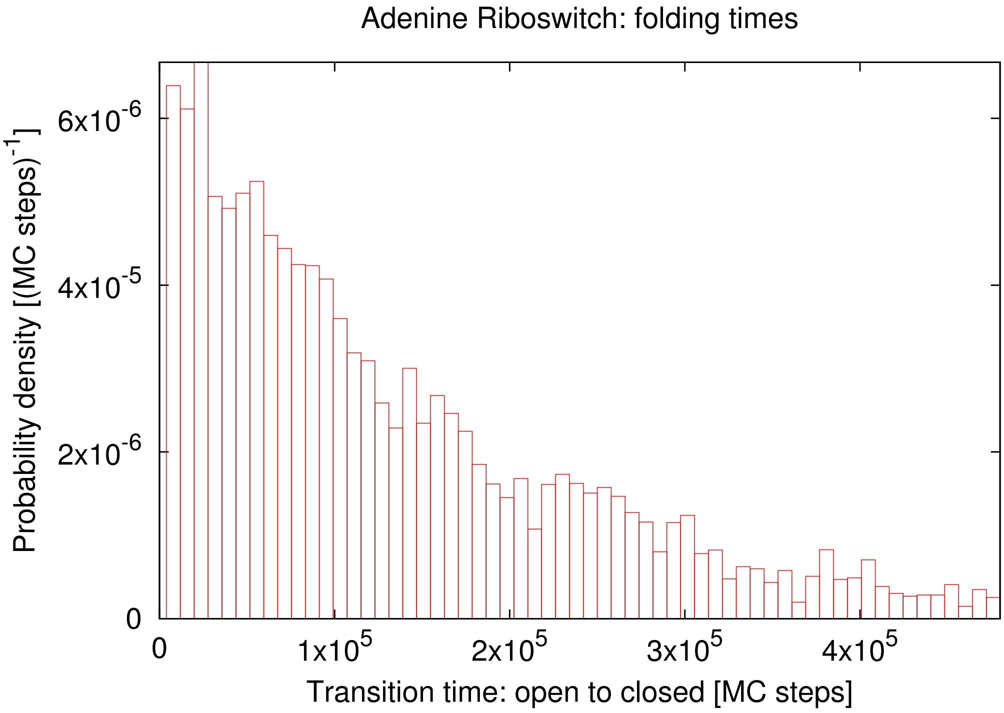

Figure 4: Histogram of folding times in kinetic MC simulations for the free folding adenine riboswitch.

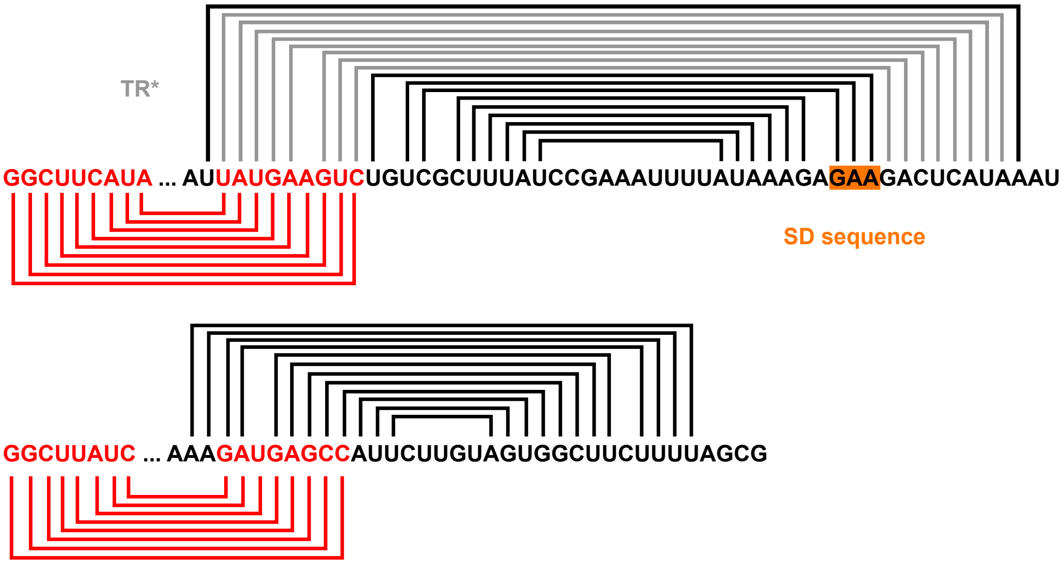

Figure 5: Secondary structure diagrams of the SAM-I (bottom) and the adenine *add* (top) riboswitch with the competing antiterminator and translational repressor (TR* directly competing with P1) conformation, respectively (black and grey).

slowly in favor of the TR (Fig 6C, "backtracking"). In fact it takes about 25 s after the whole sequence is transcribed for the TR to robustly outcompete P1. This is much slower than for the SAM-I riboswitch which can be rationalized by the fact that the adenine riboswitch acts as a translational regulator, whereas the SAM-I riboswitch directly controls transcription. The backtracking behavior can be slowed down or prevented altogether by a stabilizing ligand. We find that a stabilizing energy of $\Delta E_{multiloop} = 7\ k_BT$ is sufficient to stabilize the aptamer for long times, however lower $\Delta E_{multiloop}$ can be sufficient for the switch if the aptamer only needs to be stabilized temporarily. Recent results based on all-atom standard MD simulations give an estimate for the ligand contribution to P1 stem formation of approximately 1.6 k_BT[1] for the adenine riboswitch which is similar to the energies that we observe in our study required to stabilize the adenine aptamer for co-transcriptional folding.

3.3 Single Hairpin Folding Discussion

We observe a discrepancy in the transcription rates at which the transitions of folding orders occur. This discrepancy originates from a difference in modelling single hairpin formation. As a consequence, the folding time of an isolated single hairpin in the SBM differs from the same hairpin embedded in the whole structure, whereas in the kinetic MC simulation it does not. A histogram of the folding times of isolated helix P2 in the adenine riboswitch is shown in Fig. 8. Comparison with experimental values yields an estimate of the folding time of 0.0175 seconds for the SBM simulations. In these simulations the surrounding chain of a hairpin loop acts with a drag on both ends of the hairpin resulting in an estimate of the folding time of 0.375 seconds based on the evaluations in Sec. 3.1. The kinetic MC approach yields an estimate of the folding time of 0.00635 seconds for both the scenarios, see Fig. 9. This folding time corresponds to the folding time for the isolated hairpin by SBM simulations. Faster single hairpin folding within the whole structure explains the higher transcription rates in the kinetic MC approach at which co-transcriptional folding starts to become no longer

[1]Di Palma, F., Colizzi, F. and Bussi, G. (2013) Ligand-induced stabilization of the aptamer terminal helix in the add adenine riboswitch. *Rna.* doi:10.1261/rna.040493.113

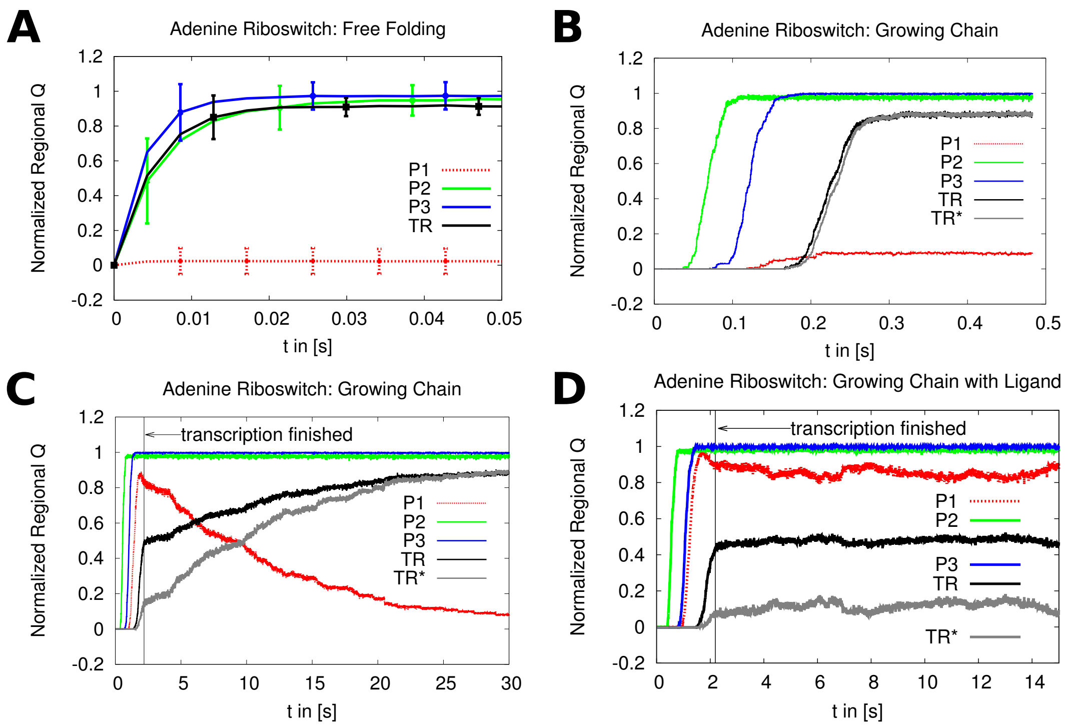

Figure 6: Folding analysis of the extended sequence of the adenine riboswitch using Kinetic MC simulations. We show the folding of substructures of the aptamer (P1, P2 and P3), the translational repressor TR and the part of the translational repressor TR*, which competes with the aptamer. (A) Normalized regional Q value as a function of time for free folding. (B) Normalized regional Q value as a function of time for the chain growing at a transcription rate of 450 nt/s. (C) Normalized regional Q value as a function of time for the chain growing at a transcription rate of 50 nt/s. (D) Normalized regional Q value as a function of time for the chain growing at a transcription rate of 50 nt/s. Here, the multiloop is stabilized by $\Delta E_{multiloop} = 7\ k_B T$.

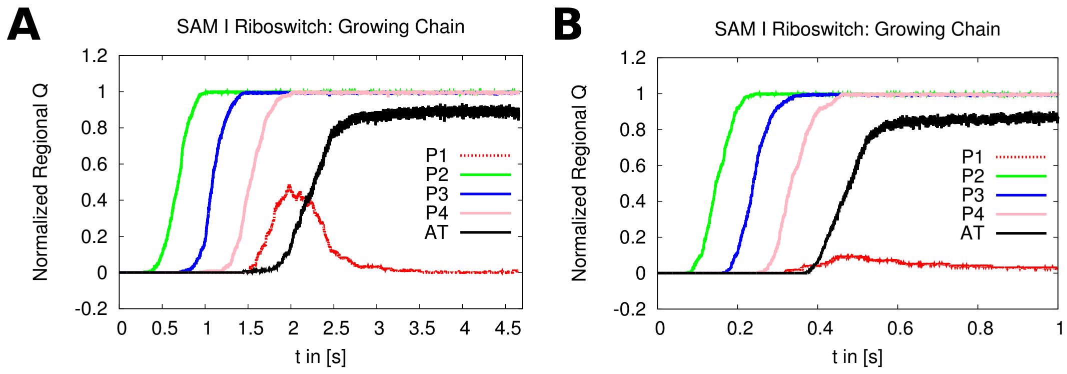

Figure 7: Possible folding pathways of the extended sequence of the SAM-I riboswitch at two different transcription rates using Kinetic MC simulations. (A) Normalized regional Q value as a function of time for the chain growing at a transcription rate of ∼50 nt/s. For transcription rates in the physiologically relevant regime backtracking of P1 can be observed. (B) Normalized regional Q value as a function of time for the chain growing at a transcription rate of ∼230 nt/s. Transcription is so fast that AT is transcribed and folds before P1 is able to form.

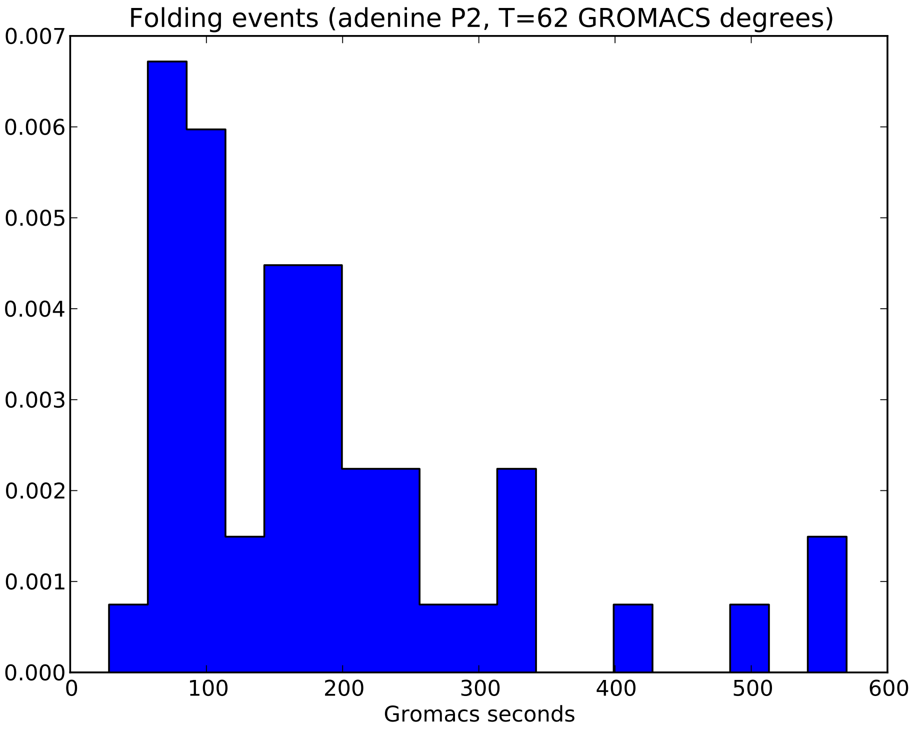

Figure 8: Normalized histogram of folding times in SBM simulations for free folding of isolated helix P2 (adenine riboswitch).

transcription rate limited.

4 Temperature Scale

SBM simulations at different temperatures are compared to a reference simulation in the AMBER forcefield at 300 K, see Fig. 10. The mean square deviations of the RMSF values, with respect to the reference simualtion, exhibit a minimum at a GROMACS/SBM temperature of 90 representing the best correpsondence, see Fig. 11.

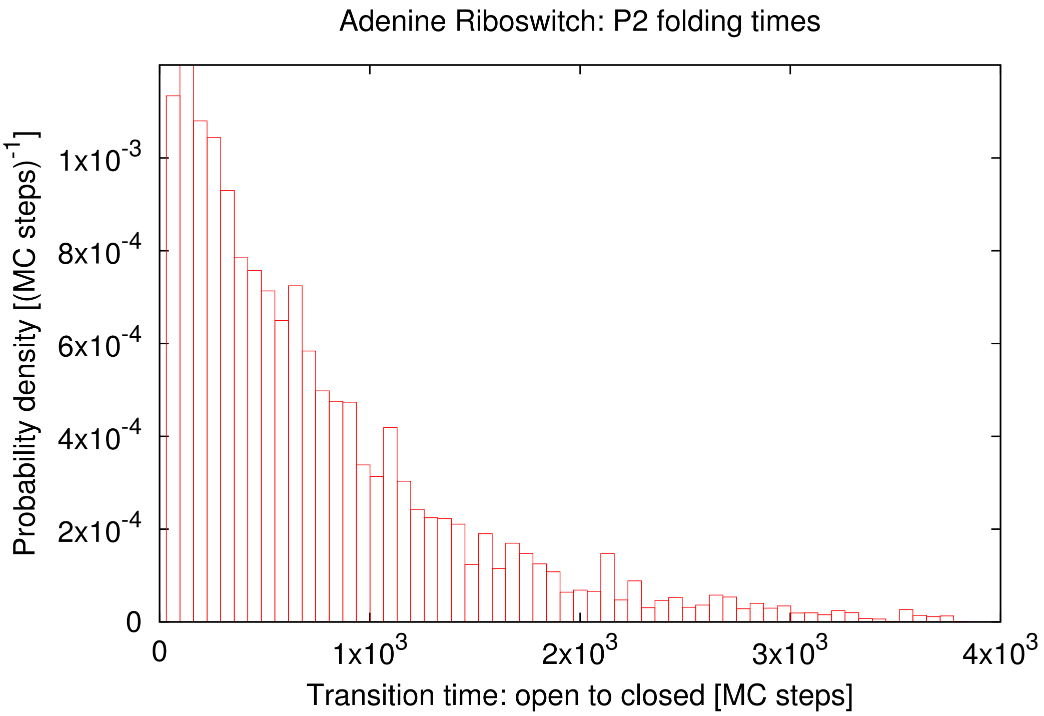

Figure 9: Histogram of folding times in kinetic MC simulations for free folding of helix P2 (adenine riboswitch).

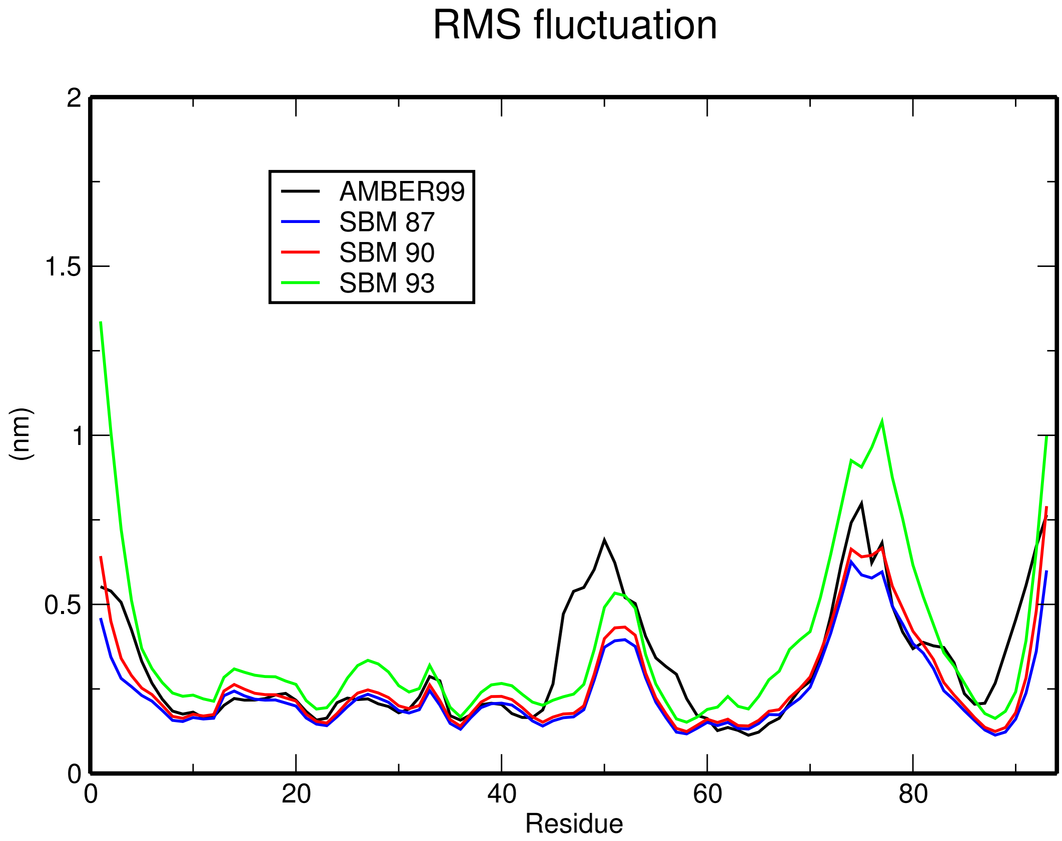

Figure 10: Root mean-square fluctuations (RMSF) of each residue by SBM and AMBER99 simulations. The SBM simulations at various temperatures can be compared with a reference simulation at 300 K in a standard molecular dynamics, all-atom, empirical AMBER99 forcefield.

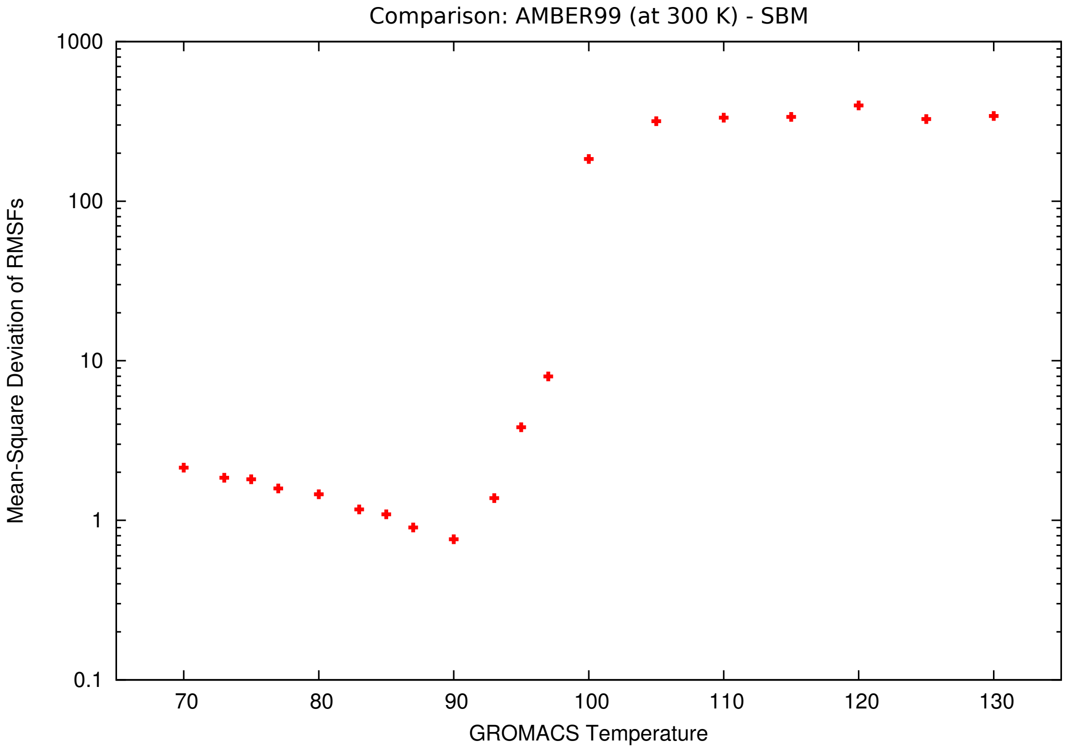

Figure 11: Mean-square deviations of the RMSFs, as seen in Fig. 10. The minimum in this plot at GROMACS/SBM temperature of 90 represents the best agreement of SBM simulation with the AMBER99 simulation at 300 K.
